# Soil Quality and Pomelo Productivity as Affected by Chicken Manure and Cow Dung

**DOI:** 10.1155/2021/6289695

**Published:** 2021-12-02

**Authors:** Le Van Dang, Ngo Phuong Ngoc, Ngo Ngoc Hung

**Affiliations:** ^1^Department of Soil Science, College of Agriculture, Can Tho University, Campus II, Can Tho 900100, Vietnam; ^2^Department of Plant Physiology-Biochemistry, College of Agriculture, Can Tho University, Campus II, Can Tho 900100, Vietnam

## Abstract

Fruit orchards in the Vietnamese Mekong Delta (VMD) are severely degraded due to many factors, such as low organic matter content, soil acidification, and poor soil management. Organic manures are considered to be a soil conservation measure that decreases soil degradation and acidity. This study aimed to evaluate the impacts of soil organic amendments on the improvement of soil fertility and pomelo productivity. Two soil amendments, namely, chicken manure (CM) and cow dung (CD), were investigated for a period of three years at three pomelo orchards. The soil quality was assessed in two depths (0–20 and 20–50 cm), including the soil pH, electrical conductivity (EC), total nitrogen (N_tot_), available phosphorus (P_avail_), soil organic matter (SOM), bulk density (BD), and exchangeable cations (Ca, Mg, and K). The results indicated that CD and CM improved soil fertility in topsoil layer (0–20 cm) due to an increase in soil pH, SOM, exchangeable Ca, N_tot_, and P_avail_. In addition, soil BD significantly reduced after CD and CM were supplied in the three consecutive years of study. The soil quality properties that significantly affected pomelo yield were SOM, N_tot_, P_avail_, and soil BD. Thus, these soil qualities may be considered as key factors for determining and assessing soil quality in fruit orchards in the VMD. More studies on the influence of organic manures on nutrient uptake and pomelo fruit quality are warranted.

## 1. Introduction

The raised bed farming technique is widely used for the alluvial soils of the VMD. It allows the cultivation of perennial crops because it avoids waterlogging during the wet season. However, there have been warnings about soil degradation in the fruit orchards wherein raised bed farming was applied [1]. Raised bed systems are constructed 1.5–2 m higher than the original ground surface, which may lead to leaching out of exchangeable cations or soil nutrients (available nitrogen (N) and potassium (P)) from the surface of colloids by rainwater during the wet season as well as irrigation during the dry season [2]. According to Quang et al. [3], soil compaction was strongly correlated with the aging of the raised beds. In addition, fruit production generally requires a high-nutrient input; thus, farmers usually supply excessive chemical fertilizers [4, 5], resulting in an increase in soil acidity [6, 7].

Pomelo is grown along the Hau and Tien rivers of the VMD and is considered to be a cash crop in the region owing to its high economy and its ability to reduce poverty among farmers [8]. Hau Giang is one of the provinces with the largest pomelo area in the VMD. However, pomelo productivity in this area tended to decrease in recent years due to land degradation [9]. A previous study revealed that soil pH, SOM, CEC, and exchangeable cations severely declined in this area [10]. Another study demonstrated that pomelo orchards have high occurrence of soil compaction and reduced soil water-holding capacity [11].

Soil quality is the capacity of soil to support ecosystem services and maintain plant productivity [12]. In agricultural systems, the functions of soil are difficult to directly assess because of its physical, chemical, and biological parameters [13, 14]. To assess the effects of land use and management on the changes in soil quality, scientists need to detect and select the most crucial soil parameters or indicators [14]. Although there are many benefits of using soil quality index as a tool in ecosystem restoration, the effectiveness of these indices may be different in soil, climate, and ecosystem types [15].

Soil amendments (biochar, compost, poultry, and cattle manure) are used for improving plant growth and productivity, which enhances soil physicochemical properties, fertility, and soil biota [16]. Various studies have reported that organic amendments improved soil organic matter, soil porosity, available water for crops, exchangeable cations, cation exchange capacity, and plant nutrients [17–19]. Poultry manure is one of the organic fertilizers that contain the most of essential nutrients for crop growth. Many studies have reported that poultry manure application improves significantly soil physical, chemical, and biological properties [20–22]. Cattle manure is an organic manure that supplies a large amount of plant nutrient and improves soil physicochemical properties when applied suitably [23]. According to Yagüe et al. [24], the application of cattle manure combined with NPK fertilizer enhanced soil organic carbon and aggregate stability compared with the application of NPK fertilizer alone.

The response of soil physicochemical properties to organic amendments may vary due to different soil groups, climates, farming techniques, and crop systems [25]. Therefore, this study aimed to evaluate the impacts of chicken manure (CM) and cow dung (CD) on the improvement of soil physicochemical properties and pomelo fruit yield cultivated in the VMD.

## 2. Materials and Methods

### 2.1. Study Site, Climate, and Soil

The research was conducted in three pomelo-growing areas of Chau Thanh District, Hau Giang Province, Vietnam: CT I, which is located at 9°54′16.4″N latitude and 105°49′48.1″E; CT II, at 9°56′51.7″N latitude and 105°45′10.8″E longitude; and CT III, at 9°51′43.8″N latitude and 105°47′20.9″E longitude. These orchards had a long history of rice cultivation after the application of raised bed farming for King mandarin plantation (about 7 years); after that, pomelos were cultivated.

The soils of study sites were classified as Gleyic Anthrosols according to the World Reference Base for Soil Resources [26]. The soil physicochemical properties of the study sites before conducting the experiments are presented in Table 1.

**Figure 1:**
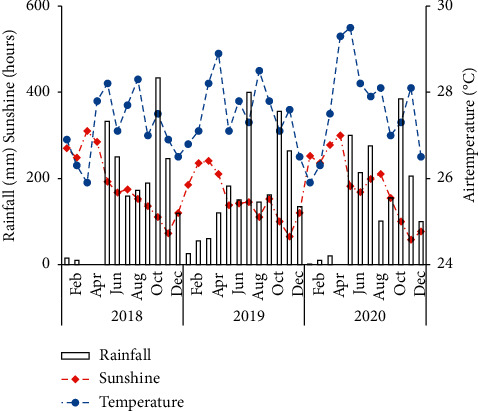
The monthly average of precipitation, temperature, and total sunshine from January 2018 to December 2020 in Hau Giang Province (source: Hau Giang Hydrometeorological Station).

The climate data in the research area during the period from 2018 to 2020 were taken from the Hau Giang Hydrometeorological Station.  Figure 1 presents the monthly mean air temperature and rainfall before the experiments were conducted.

### 2.2. Plant, Cow Dung, and Chicken Manure

The study was conducted in a six-year-old plantation of “5 Roi” pomelo grown by rootstock. The trees were spaced at 4.0 × 4.0 m. At the start of the experiment, the pomelo trees were 3.5 to 3.75 m tall and had a canopy with a size of 3.0 to 3.25 m. The trees had similar trunk diameters selected for the trial.

The CD used in this research was a commercial product of the Vinatap Viet Nam, which contained 394 g of total C kg^−1^, 15.7 g of total N kg^−1^, 9.85 g of total P kg^−1^, and 8.96 g of total K kg^−1^. Chicken manure was incubated between raw CM with rice straw for 60 days under field conditions. When starting incubation, raw CM and rice straw irrigated gain from 55% to 60% humidity. The CM contained 475 g of total C kg^−1^, and the contents of macroelements, namely, N, P, and K, were 19.6, 9.07, and 24.7 g kg^−1^, respectively.

### 2.3. Experimental Design

The experiment was conducted in a randomized complete block design with four replications of three treatments as follows: control, only applied NPK fertilizer; cow dung (CD) applied at 10 Mg per year per ha combined with NPK fertilizer; chicken manure (CM) applied at 10 Mg per year per ha combined with NPK fertilizer. The study was conducted for three consecutive years, from January 2018 to December 2020. A total of 48 pomelo trees were used per experiment per site; each treatment included 16 trees, and each replicate was four trees. CM and CD were applied twice per year (depth of about 10 cm from the surface layer around pomelo canopy), at the start and at the end of the dry season. All treatments in this study accepted normal horticultural care for pest, weed, and disease control.

The application rates of N, P, and K (900, 600, and 850 g per tree per year, resp.) were in accordance with the recommendation of the Southern Horticultural Research Institute (SOFRI), Vietnam. N, P, and K were applied as urea (46% N), superphosphate (7% P), and potassium chloride (50% K). After one month of the harvested stage, 20% of total N and 30% of total P were applied; 15%, 40%, and 30% of total N, P, and K, respectively, were applied before pomelo blossomed at two months; and 20%, 10%, and 15% of total N, P, and K, respectively, were applied at one month after fruit set. At two and a half months after fruit set, 25%, 10%, and 15% of total N, total P, and total K were applied for pomelo tree, and at four months after fruit set, 20%, 10%, and 20%, respectively, were applied; 20% of total K was applied two months before fruit harvest.

### 2.4. Soil Sampling and Analysis

Soil sample collection was performed in December 2018, 2019, and 2020. The soil samples were taken from two layers, surface (0–20-cm depth) and subsurface (20–50-cm depth), at five different positions for each replicate and then mixed evenly to obtain a soil sample. At the same time, the separate soil samples were taken with 100 cm^3^ cores for bulk density (BD) analysis. After being collected, the soil samples were put in plastic bags and then transported to the Soil Physics and Chemistry Laboratory, Department of Soil Science, College of Agriculture, Can Tho University. In our study, a total of 216 soil samples were collected in three years from three sites (72 samples each year).

To analyze the soil chemical parameters, the soil samples were air-dried at 25°C–28°C for 10 days, crushed and sieved through 0.5 and 2.0 mm mesh, and stored in the plastic box. The soil physicochemical properties (pH, EC, SOM, N_tot_, P_avail_, BD, Ca^2+^, Mg^2+^, and K^+^) were analyzed according to the standard procedures described by Houba et al. [27]. Soil pH in the 1 : 2.5 solution (soil/water) was determined using a digital pH meter, and EC was determined using a digital conductivity meter. SOM was determined using the Walkley–Black method [28], N_tot_ using the Kjeldahl method [29], and P_avail_ using the Bray II method [30]. BD was determined using the core method; soil cores were oven-dried at 105°C, and BD was calculated as mass of oven-dried soil divided by the total volume [31]. Exchangeable cations (Ca^2+^, Mg^2+^, and K^+^) were extracted with 0.1 M BaCl_2_ solution and measured via flame photometry [32].

### 2.5. Pomelo Yield and Data Analysis

The productivity of pomelo (t ha^−1^) was calculated as fruit weight total per tree multiplied by plant density. The fruits were harvested three times per year. In this study, we used the SPSS software (version 16.0) for data statistics. The mean values were calculated via analysis of variance, and comparison of the differences between the treatments was performed using Duncan's post hoc test at *p* < 0.05. Principal component analysis (PCA) was conducted on the soil characteristics to divide the variables with a high percentage of correlation. Regression analysis was employed to determine the relationship between soil physicochemical properties and fruit yield.

## 3. Results and Discussion

### 3.1. Effects of Organic Amendments on Soil Physicochemical Properties

#### 3.1.1. Topsoil Layer (0–20 cm)

The application of CM and CD significantly improved pH, SOM, exchangeable Ca, BD, N_tot_, and P_avail_ compared with the control (Table 2). It also significantly increased soil pH in the three study sites. The soil pH was significantly greater with CM than with CD in CT I and CT II. Contrarily, soil pH was higher with CD than with CM in CT III. Soil EC and exchangeable K were not affected by the application of CD and CM in the study sites. A significant difference in SOM was observed after the application of CD and CM. Similarly, exchangeable Ca was significantly greater with CD and CM than with the control treatment in the study sites. However, the application of CD and CM did not enhance the concentration of exchangeable Mg in this study, except for CT III. In our research, soil BD was greatly decreased by the application of CD and CM; moreover, soil BD significantly decreased with CD than with CM in CT II and CT III. The N_tot_ and P_avail_ contents increased after the application of CD or CM.

Many previous studies reported that the application of CM or CD improved soil fertility and decreased the occurrence of soil compaction [33–35]. According to Adekiya et al. [33], soil pH significantly increased after the application of organic amendments due to the release of calcium ions into the soil solution during the microbial decarboxylation of manure. In this study, the concentration of exchangeable Ca was significantly elevated by CD and CM application. Furthermore, a positive correlation was observed between soil pH and Ca in the topsoil and subsoil layers (Tables 3 and 4). In our research, the contents of SOM, N_tot_, and P_avail_ were significantly increased by the application of both CD and CM. The results are in agreement with the findings of Adebayo et al. [36] and Yunilasari et al. [34]. The CM and CD used in the experiment contained high total organic carbon and N and P nutrients (Section 2.2) that may increase the concentration of N_tot_ and P_avail_ compared with the control. We observed a significant decrease in BD with the application of 10 Mg ha^−1^ year^−1^ of CD or CM in our three-year field experiment (Table 2). Guo et al. [37] and Adekiya et al. [38] also elucidated that the decrease in soil BD might be associated with soil organic carbon, which is highly contained in animal manure.

A positive correlation was observed between the soil quality parameters in topsoil horizon (Table 3), such as the following: SOM and Ca (*r* = 0.48), SOM and K (*r* = 0.47), SOM and Mg (*r* = 0.70), SOM and N_tot_ (*r* = 0.93), and SOM and P_avail_ (*r* = 0.87). Contrarily, SOM had a strong negative correlation with soil BD (*r* = −0.80). Soil pH, Ca^2+^, K^+^, Mg^2+^, N_tot_, and P_avail_ were also negatively correlated with soil BD (*r* = −0.30, *r* = −0.45, *r* = −0.28, *r* = −0.53, *r* = −0.76, and *r* = −0.73, resp.). A positive correlation was observed between P_avail_ and N_tot_ (*r* = 0.89), P_avail_ and exchangeable Ca (*r* = 0.57), P_avail_ and exchangeable K (*r* = 0.45), and P_avail_ and exchangeable Mg (*r* = 0.63). Similarly to P_avail_, N_tot_ was positively correlated with Ca^2+^ (*r* = 0.53), K^+^ (*r* = 0.43), and Mg^2+^ (*r* = 0.73). Soil pH was positively correlated with exchangeable Ca (*r* = 0.40). According to Khadka et al. [39], there was a positive correlation between soil pH and exchangeable Ca. Furthermore, Dang et al. [40] confirmed that soil BD was negatively correlated with pH, SOM, and exchangeable cations (Ca^2+^and Mg^2+^), at *r* values of −0.72, −0.66, −0.81, and −0.75, respectively. Wibowo and Kasno [41] indicated that soil organic carbon and total nitrogen had a strong positive correlation. Yang et al. [42] concluded that soil available phosphorus increased when the SOM content increased.

#### 3.1.2. Subsoil Layer (20–50 cm)

All the soil physicochemical properties at the 20–50-cm depth were not improved by the application of CM and CD, except for pH, exchangeable Ca and Mg at CT III and pH at CT I (Table 5). Compared with the control, CM application in the CT III location increased pH by about 0.45 unit and the exchangeable cations, namely, Ca and Mg, by 0.89 and 0.63 meq 100g^−1^, respectively. Meanwhile, the application of CD increased pH, Ca^2+^, and Mg^2+^ by 0.33 units, 0.88 meq 100g^−1^, and 0.56 meq 100g^−1^. These results are in accordance with those of Canali et al. [43] who reported no difference in the soil properties after a long-term addition of composts and poultry manure. In this study, CM and CD were applied in soil at a depth of only about 10 cm from the topsoil layer. In addition, the short duration of the experiment may also cause a decrease in the impact of organic manure on soil quality. A similar result has been reported by Mokgolo et al. [44]. Rees et al. [45] indicated that the application of CM improved the soil biological properties but did not change the soil organic carbon content and soil physical properties in the three-year study duration.

Table 4 demonstrates that soil EC was not significantly related to other physicochemical properties, except for exchangeable Ca (*r* = −0.26). Similar to the topsoil layer, a negative correlation was observed between SOM and BD (*r* = −0.50), K^+^ and BD (*r* = −0.30), Mg^2+^ and BD (*r* = −0.28), N_tot_ and BD (*r* = −0.36), and P_avail_ and BD (*r* = −0.30). Furthermore, exchangeable Mg was positively correlated with SOM, exchangeable Ca, and K (*r* = 0.23, *r* = 0.33, and *r* = 0.42, resp.). Soil pH was negatively correlated with P_avail_ (*r* = −0.23) and positively correlated with exchangeable Ca (*r* = 0.31). Available P was positively correlated with K^+^ (*r* = 0.18) and negatively correlated with total N (*r* = −0.30). Tables 3 and 4 show that the relationship between soil quality properties is complex.

#### 3.1.3. Fruit Yield

**Figure 2:**
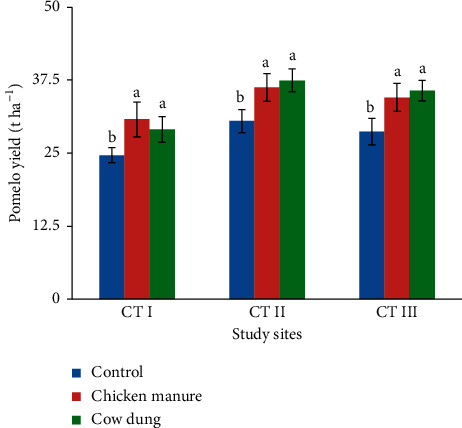
Pomelo yield affected by chicken manure and cow dung (mean value: 2018–2021). The different letters indicate the significant differences among treatments at *p* < 0.001. Error bars in the column represent standard deviation.

A significant difference (*p* < 0.001) was observed in pomelo yield in the CM and CD treatments compared with the control in the three study sites (Figure 2). Contrarily, no difference in pomelo productivity was observed between the CD and CM treatments in the study sites. The lowest amount of fruit yield was observed in the control treatment. The highest yield was observed in the CM or CD application at a dose of 10 Mg ha^−1^ year^−1^. Compared with the control, CM increased pomelo yield by 6.13, 5.75, and 5.88 t ha^−1^, whereas CD increased the yield by 4.39, 6.98, and 7.02 t ha^−1^ in CT I, CT II, and CT III locations, respectively.

**Figure 3:**
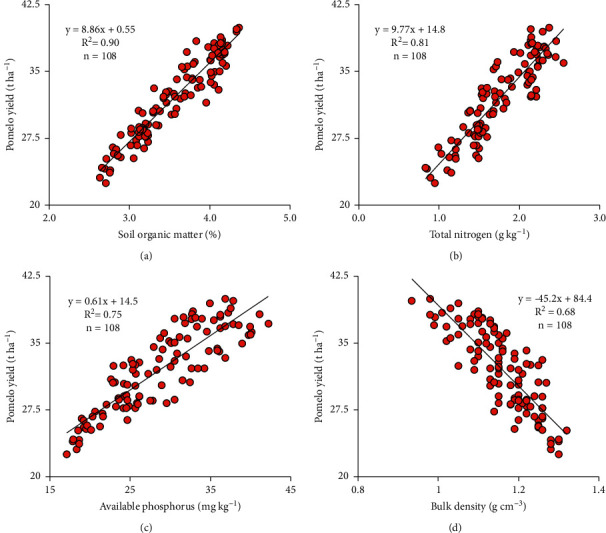
Relationship between fruit yield and the four soil quality parameters at a depth of 0–20 cm.

According to Akosah et al. [46], the application of CM reduced fruit drop and enhanced sweet orange fruit yield. In our study, the use of CD and CM increased the soil nutrients (SOM, N_tot_, and P_avail_) that have a strong positive correlation with the pomelo yield (Figure 3), thus enhancing fruit yield. This result is in agreement with the results of Timsina [47] who reported that the release of nutrients during the decay processes of animal manure improved crop yield. Similar results have also been reported by Eissa [48], Yunilasari et al. [34], and Adebayo et al. [49].

### 3.2. The Relationship between Soil Physicochemical Characteristics and Fruit Yield

In our study, PCA was grouped into three components, with 80.8% of the total variance explained (Table 6). PC1 included soil quality properties, such as SOM, exchangeable cations (Ca, K, and Mg), soil nutrients (N_tot_ and P_avail_), soil physical property (BD), and fruit yield. The parameters of exchangeable cations presented values above 0.50, whereas SOM and soil nutrients had coefficients over 0.90. About 55.5% of the total variance was explained by this component. PC2 and PC3 included soil pH and EC, respectively. The percentages of variance interpreted were about 14.9% and 15.2% in these two components, respectively. According to Ghaemi et al. [50], PCA is widely used for soil quality assessment because it creates a linear combination of input data. In addition, it is used to calculate scores for soil quality indices [51].

The regression analysis in Figure 3 shows a positive correlation between the productivity of fruit and SOM (*y* = 8.86*x* + 0.55; *R*^2^ = 0.90) and total nitrogen (*y* = 9.77*x* + 14.8; *R*^2^ = 0.81) and available phosphorus (*y* = 0.61*x* + 14.5; *R*^2^ = 0.75). Pomelo yield and soil BD had a negative correlation, which is expressed as *y* = −45.2*x* + 84.4 (*R*^2^ = 0.68). These results indicate that improvements in soil nutrients positively affected pomelo yield. This indicates that pomelo productivity tends to increase with the increase in the soil quality parameters (SOM, N_tot_, and P_avail_). Similar results were obtained by Sainju et al. [52] and Liu et al. [53], who found significant positive correlation coefficients between soil nutrients (organic carbon, N, and P) and plant yield. An increase in soil BD is known to decrease root penetration and nutrient uptake as well as root formation reduction, which may decrease crop yield [54].

## 4. Conclusion

The results indicated that organic manures, such as CD and CM, play a vital role in the improvement of soil fertility and decrease in soil degradation, thus enhancing pomelo fruit yield compared with the use of inorganic fertilizer alone. Regression and PCA indicated that SOM, total nitrogen, and available phosphorus were closely related to pomelo productivity with high determination coefficients. Therefore, these parameters were selected as soil quality indicators for evaluating soil productivity. From the results of this study, we recommend the use of CD and CM as the best choice for sustainable agriculture. However, further studies are necessary to assess the effects of CD and CM on nutritional status and pomelo fruit quality.

## Figures and Tables

**Table 1:** Initial physicochemical properties of the experimental soils.

Parameters	Unit	CT I		CT II		CT III	
		0–20 cm	20–50 cm	0–20 cm	20–50 cm	0–20 cm	20–50 cm
pH_H2O_		5.19	5.07	4.34	4.83	4.68	5.01
EC	mS cm^−1^	0.77	0.70	0.79	0.65	0.71	0.62
Available phosphorus	mg kg^−1^	18.9	22.5	25.8	19.6	32.1	16.5
Total nitrogen	g kg^−1^	0.91	1.32	1.61	1.05	1.52	1.96
Soil organic matter	%	2.87	2.83	3.56	3.35	3.37	3.45

*Exchangeable cations*							
Na^+^	meq 100g^−1^	0.26	0.31	0.06	0.13	0.06	0.10
K^+^		0.24	0.12	0.40	0.22	0.20	0.16
Ca^2+^		5.73	6.52	5.80	6.39	6.90	6.83
Mg^2+^		1.94	2.22	2.91	3.16	2.79	2.66
CEC	meq 100g^−1^	19.9	19.3	20.6	21.0	21.5	20.4
Bulk density	g cm^−3^	1.25	1.27	1.22	1.20	1.18	1.11

*Soil texture*							
Sand	%	1.00	1.50	0.70	1.80	1.90	0.40
Silt		46.5	40.2	48.6	35.1	44.9	50.5
Clay		52.5	58.3	50.7	63.1	53.2	49.1

*Soil textural class*		Silty clay	Silty clay	Silty clay	Clay	Silty clay	Silty clay

**Table 2:** Influence of animal manure on soil quality in the surface layer (mean value: 2018–2020).

Sites	Treatments	pH_H2O_ (1 : 2.5)	EC (mS cm^−1^)	SOM (%)	Exchangeable cations (meq 100g^−1^)			BD (g cm^−3^)	N_tot_ (g kg^−1^)	P_avail_ (mg kg^−1^)
					Ca^2+^	K^+^	Mg^2+^			
CT I	Control	5.12^c^ ± 0.17	0.71 ± 0.09	2.75^b^ ± 0.08	5.81^b^ ± 0.45	0.27 ± 0.05	2.20 ± 0.30	1.27^b^ ± 0.03	1.05^b^ ± 0.14	18.6^c^ ± 0.74
	CM	6.00^a^ ± 0.25	0.75 ± 0.07	3.34^a^ ± 0.26	6.50^a^ ± 0.30	0.32 ± 0.07	2.84 ± 0.55	1.13^a^ ± 0.07	1.47^a^ ± 0.21	24.3^b^ ± 3.10
	CD	5.75^b^ ± 0.15	0.77 ± 0.10	3.24^a^ ± 0.25	6.48^a^ ± 0.17	0.29 ± 0.09	2.96 ± 1.00	1.18^a^ ± 0.05	1.56^a^ ± 0.12	24.8^a^ ± 1.75
	P_value_	^*∗∗∗*^	ns	^*∗∗∗*^	^*∗∗∗*^	ns	ns	^*∗∗∗*^	^*∗∗∗*^	^*∗∗∗*^

CT II	Control	4.20^c^ ± 0.13	0.76 ± 0.07	3.40^c^ ± 0.24	5.78^b^ ± 0.28	0.40 ± 0.09	3.32 ± 0.13	1.24^c^ ± 0.03	1.56^b^ ± 0.13	25.1^b^ ± 1.15
	CM	4.91^b^ ± 0.15	0.71 ± 0.10	3.97^b^ ± 0.19	6.22^a^ ± 0.70	0.44 ± 0.06	3.97 ± 0.50	1.12^b^ ± 0.05	2.13^a^ ± 0.21	32.6^a^ ± 1.97
	CD	5.14^a^ ± 0.30	0.70 ± 0.05	4.18^a^ ± 0.11	6.52^a^ ± 0.23	0.37 ± 0.07	3.82 ± 0.89	1.05^a^ ± 0.07	2.19^a^ ± 0.08	33.7^a^ ± 3.34
	P_value_	^*∗∗∗*^	ns	^*∗∗∗*^	^*∗∗*^	ns	ns	^*∗∗∗*^	^*∗∗∗*^	^*∗∗∗*^

CT III	Control	4.66^c^ ± 0.27	0.73 ± 0.06	3.23^c^ ± 0.23	6.55^b^ ± 0.36	0.36 ± 0.06	3.05^b^ ± 0.32	1.21^c^ ± 0.03	1.54^b^ ± 0.08	29.8^c^ ± 4.34
	CM	5.56^a^ ± 0.24	0.69 ± 0.09	3.81^b^ ± 0.19	7.10^a^ ± 0.46	0.36 ± 0.06	3.81^a^ ± 0.18	1.14^b^ ± 0.02	2.18^a^ ± 0.13	35.9^b^ ± 3.54
	CD	5.24^b^ ± 0.19	0.69 ± 0.06	4.03^a^ ± 0.18	7.14^a^ ± 0.15	0.39 ± 0.06	3.73^a^ ± 0.21	1.10^a^ ± 0.06	2.11^a^ ± 0.22	38.5^a^ ± 3.06
	P_value_	^*∗∗∗*^	ns	^*∗∗∗*^	^*∗∗*^	ns	^*∗∗*^	^*∗∗∗*^	^*∗∗∗*^	^*∗∗∗*^

The different letters indicate the significant differences among treatments at *p* < 0.01 (^*∗∗*^) and *p* < 0.001 (^*∗∗∗*^); ns: not significant; CM: chicken manure applied at 10 mg per year; CD: cow dung applied at 10 mg per year. CT I, CT II, and CT III are the study locations.

**Table 3:** Matrix correlation between soil physicochemical properties at a depth of 0–20 cm (*n* *=* 108).

	pH	EC	SOM	Ca^2+^	K^+^	Mg^2+^	BD	N_tot_	P_avail_
pH	1	−0.03	0.01	0.40^*∗∗∗*^	−0.30^*∗∗*^	−0.03	−0.30^*∗∗*^	0.04	0.03
EC		1	−0.13	−0.01	−0.04	0.02	0.18^*∗*^	−0.20^*∗*^	−0.27^*∗*^
SOM			1	0.48^*∗∗∗*^	0.47^*∗∗∗*^	0.70^*∗∗∗*^	−0.80^*∗∗∗*^	0.93^*∗∗∗*^	0.87^*∗∗∗*^
Ca^2+^				1	0.12	0.39^*∗∗∗*^	−0.45^*∗∗∗*^	0.53^*∗∗∗*^	0.57^*∗∗∗*^
K^+^					1	0.37^*∗∗∗*^	−0.28^*∗∗*^	0.43^*∗∗∗*^	0.45^*∗∗∗*^
Mg^2+^						1	−0.53^*∗∗∗*^	0.73^*∗∗∗*^	0.63^*∗∗∗*^
BD							1	−0.76^*∗∗∗*^	−0.73^*∗∗∗*^
N_tot_								1	0.89^*∗∗∗*^
P_avail_									1

^*∗*^, ^*∗∗*^, and ^*∗∗∗*^ indicate significant difference at *p* < 0.05, *p* < 0.01, and *p* < 0.001, respectively.

**Table 4:** Matrix correlation between soil physicochemical properties at a depth of 20–50 cm (*n* *=* 108).

	pH	EC	SOM	Ca^2+^	K^+^	Mg^2+^	BD	N_tot_	P_avail_
pH	1	0.16	−0.12	0.31^*∗∗*^	0.01	−0.08	0.29^*∗∗*^	0.14	−0.23^*∗∗*^
EC		1	0.08	−0.26^*∗*^	−0.06	0.03	0.01	0.03	−0.10
SOM			1	−0.17^*∗*^	−0.22^*∗*^	0.23^*∗∗*^	−0.50^*∗∗∗*^	−0.01	−0.09
Ca^2+^				1	0.11	0.33^*∗∗*^	0.25^*∗∗*^	−0.01	−0.12
K^+^					1	0.42^*∗∗∗*^	0.34^*∗∗∗*^	−0.30^*∗∗*^	0.18^*∗*^
Mg^2+^						1	0.10	−0.28^*∗∗*^	0.005
BD							1	−0.36^*∗∗∗*^	0.11
N_tot_								1	−0.30^*∗∗*^
P_avail_									1

^*∗*^, ^*∗∗*^, and ^*∗∗∗*^ indicate significant difference at *p* < 0.05, *p* < 0.01, and *p* < 0.001, respectively.

**Table 5:** Impacts of chicken manure and cow dung on soil physicochemical properties in the subsurface layer (mean value: 2018–2020).

Sites	Treatments	pH_H2O_ (1 : 2.5)	EC (mS cm^−1^)	SOM (%)	Exchangeable cations (meq 100g^−1^)			BD (g cm^−3^)	N_tot_ (g kg^−1^)	P_avail_ (mg kg^−1^)
					Ca^2+^	K^+^	Mg^2+^			
CT I	Control	4.80^b^ ± 0.29	0.70 ± 0.05	2.73 ± 0.34	6.37 ± 0.52	0.32 ± 0.13	2.70 ± 1.16	1.31 ± 0.10	1.55 ± 0.16	22.9 ± 3.13
	CM	5.29^a^ ± 0.30	0.75 ± 0.08	2.79 ± 0.29	7.03 ± 1.81	0.27 ± 0.10	2.78 ± 0.39	1.25 ± 0.10	1.58 ± 0.17	22.5 ± 2.20
	CD	5.17^a^ ± 0.49	0.76 ± 0.09	2.85 ± 0.84	6.96 ± 1.23	0.34 ± 0.10	2.66 ± 0.70	1.29 ± 0.17	1.67 ± 0.18	23.8 ± 3.18
	P_value_	^*∗*^	ns	ns	ns	ns	ns	ns	ns	ns

CT II	Control	4.91 ± 0.24	0.76 ± 0.05	3.72 ± 0.37	6.52 ± 1.88	0.29 ± 0.12	3.36 ± 1.63	1.26 ± 0.17	1.16 ± 0.16	21.6 ± 2.06
	CM	5.15 ± 0.55	0.79 ± 0.10	3.64 ± 0.39	7.23 ± 1.74	0.33 ± 0.14	4.23 ± 1.64	1.27 ± 0.11	1.07 ± 0.17	22.1 ± 2.33
	CD	5.24 ± 0.74	0.82 ± 0.12	3.68 ± 0.48	7.29 ± 0.98	0.29 ± 0.11	4.37 ± 1.39	1.29 ± 0.15	1.23 ± 0.15	22.9 ± 2.80
	P_value_	ns	ns	ns	ns	ns	ns	ns	ns	ns

CT III	Control	5.06^b^ ± 0.56	0.84 ± 0.13	3.36 ± 0.52	6.72^b^ ± 0.65	0.21 ± 0.03	2.79^b^ ± 0.42	1.19 ± 0.11	1.84 ± 0.11	20.4 ± 1.85
	CM	5.51^a^ ± 0.16	0.79 ± 0.09	3.28 ± 0.23	7.61^a^ ± 0.71	0.21 ± 0.05	3.42^a^ ± 0.46	1.21 ± 0.09	1.88 ± 0.13	19.5 ± 2.24
	CD	5.39^a^ ± 0.21	0.76 ± 0.07	3.44 ± 0.15	7.60^a^ ± 0.50	0.23 ± 0.08	3.35^a^ ± 0.25	1.19 ± 0.06	1.92 ± 0.19	18.7 ± 1.80
	P_value_	^*∗*^	ns	ns	^*∗∗∗*^	ns	^*∗∗*^	ns	ns	ns

The different letters indicate the significant differences among treatments at *p* < 0.05 (^*∗*^), *p* < 0.01 (^*∗∗*^), and *p* < 0.001 (^*∗∗∗*^); ns: not significant; CM: chicken manure applied at 10 mg per year; CD: cow dung applied at 10 mg per year. CT I, CT II, and CT III are the study locations.

**Table 6:** Principal component matrix in the topsoil layer (0–20 cm).

Principal components	PC1	PC2	PC3
pH_H2O_ (1 : 2.5)	0.096	0.902	0.018
EC (mS cm^−1^)	−0.195	−0.033	0.957
Soil organic matter (%)	0.958	−0.081	0.031
Exchangeable Ca^2+^ (meq 100g^−1^)	0.589	0.505	0.206
Exchangeable K^+^ (meq 100g^−1^)	0.508	−0.585	0.071
Exchangeable Mg^2+^ (meq 100g^−1^)	0.762	−0.133	0.259
Bulk density (g cm^−3^)	−0.843	−0.225	0.067
Total nitrogen (g kg^−1^)	0.955	−0.025	−0.019
Available phosphorus (mg kg^−1^)	0.929	−0.021	−0.111
Fruit yield (t ha^−1^)	0.953	−0.064	−0.024
Total	5.54	1.49	1.05
Percentage of variance	55.4	14.9	10.5
Cumulative percentage variance	55.5	70.4	80.6

## Data Availability

All data supporting the conclusions of this study are included in this article.
